# Recombinant human bone morphogenetic protein 2 and 7 inhibit the degeneration of intervertebral discs by blocking the Puma-dependent apoptotic signaling

**DOI:** 10.7150/ijbs.56823

**Published:** 2021-06-11

**Authors:** Shiwei Xie, Chenyang Zhao, Wei Chen, Gengwu Li, Zhiwei Xiong, Xiangjun Tang, Fan Zhang, Heng Xiao

**Affiliations:** 1 Department of Orthopaedics, Panzhihua Central Hospital, Panzhihua City, Sichuan, 617067, China.; 2 Department of Orthopedics, The First Affiliated Hospital of Kunming Medical University, Kunming 650032, Yunan, China.

**Keywords:** BMP, Puma, IDD, Smad1/5/8, Smad4

## Abstract

Recombinant human bone morphogenetic proteins (rhBMPs) can stimulate bone formation and growth in the treatment of spinal fusions and nonunions. However, it is still unclear whether rhBMPs function in the prevention of intervertebral disc degeneration (IDD). Here, we discovered that BMP levels were decreased in IDD patients, which impaired the BMP/Smad (Mothers against decapentaplegic homologs) signaling. Conducting a microarray assay in Smad4-knockdown cells, we found that expression of *PUMA* (p53-upregulated modulator of apoptosis) was significantly induced. The molecular analysis revealed that Smad4 recruited HDAC1 (histone deacetylase 1) and the phosphorylated Smad1/5/8 to dock on the promoter of PUMA to repress its expression. The impairment of BMP/Smad signaling in IDD patients caused the significant induction of Puma-dependent apoptosis and resulted in the pathogenesis of IDD. *In vitro* knockdown of BMP receptors (BMPR1a and BMPR2) in nucleus pulposus (NP) cells could mimic the molecular changes of BMP/Smad signaling and Puma-dependent apoptotic signaling that were observed in IDD patients. Exposing NP cells to RITA (reactivating p53 and inducing tumor apoptosis) small molecule and rhBMP2 (or rhBMP7), we observed that rhBMP2/7 could significantly decrease protein levels of Puma and its downstream proapoptotic molecules, blocking cell apoptosis. Importantly, administration of rhBMPs in aged rats could inhibit the occurrence of IDD. Our results provide a link between BMP/Smad signaling and Puma-dependent apoptotic signaling, revealing a new mechanism of how BMPs contribute to IDD pathogenesis and providing evidence that rhBMPs may decrease apoptosis and improve the outcome of IDD.

## Introduction

The intervertebral disc (IVD) is a fibrocartilaginous joint that consists of three major components: nucleus pulposus (NP), annulus fibrosus (AF), and cartilaginous endplates 1,2. With aging, the nucleus and annulus tissues undergo degenerative changes, cause the destruction of endplate cartilage, and lose their cushioning ability, leading to intervertebral disc degeneration (IDD) 3,4. IDD is an inevitable process, and it is ubiquitous among patients with low back pain (LBP) 5. According to statistical data from the World Health Organization, nearly 80% of the population experiences LBP throughout their lifetime, which significantly increases the socioeconomic burden 6. In-depth study of the pathogenic mechanisms of IDD will benefit its treatment and even delay its pathological process.

Apoptosis is considered to be one of the major mechanisms that cause IDD 7. Apoptosis is a highly regulated process, and it is mainly initiated through two pathways: intrinsic and extrinsic pathways 8. The intrinsic pathway is activated by both exogenous and endogenous stresses, such as hypoxia, DNA damage, and survival factor deprivation 9. A representative symptom of the intrinsic pathway is the permeabilization of mitochondria and the release of cytochrome *c* 10. The permeabilization of mitochondria is controlled by several BH3-only proteins known as BID (BH3-interacting domain death agonist), BIM (BH3-interacting mediator of cell death), and Puma (p53-upregulated modulator of apoptosis) 11,12. These proteins, together with BAX (BCL2-associated protein X) and BAK (BCL2-antagonist/killer), induce conformational changes of the mitochondria 11-13. Cytochrome *c* releases from the mitochondria into the cytoplasm, where it binds to Apaf-1 (apoptotic protease-activating factor-1) 11-13. Apaf-1 recruits caspase-9 to assemble the apoptosome, which cleaves pro-caspase-9 to the active caspase-9, and the latter further triggers the cleavage of pro-caspase-3 11-13. The extrinsic pathway is initiated by a death ligand (e.g., TNF-α [tumor necrosis factor-alpha], TRAIL [TNF-related apoptosis-inducing ligand], and FasL [Fas ligand]) binding to a death receptor (e.g., TNFR1 [TNF-α receptor 1], DR4/5 [death receptor 4 and 5], and FGFR [fibroblast growth factor receptor]) 14-16. Once a death ligand binds to its receptor on the cell membrane, the receptors recruit adaptor proteins such as FADD (Fas-associated protein with death domain) and TRADD (TNFR1-associated DEATH domain protein) 14-16. The adaptor proteins recruit a series of downstream factors, such as caspase-8 and caspase-10, which trigger apoptosis directly by cleaving and activating executioner caspase-3/6/7 14-16. Although the activation of both intrinsic and extrinsic pathways has been observed in the pathogenesis of IDD, it is still unclear how they are initiated and if they crosslink with other signaling pathways.

Bone morphogenetic proteins (BMPs) are a subclass of transforming growth factor-beta (TGF-β) proteins 17,18. They play essential roles in the formation and maintenance of bone, cartilage, and muscle 19,20. BMPs elicit their effects through binding to two types of serine-threonine kinase transmembrane receptors, known as BMPRI and BMPRII 17-20. Once the receptors are activated, they phosphorylate three Mothers against decapentaplegic homologs (Smads) - Smad1, Smad5, and Smad8 - at the S-S-X-S motifs located in their carboxy-terminals 17-20. These Smads are called receptor-regulated Smads (R-Smads) 17-20. The phosphorylated Smad1/5/8 assemble a complex with the unphosphorylated Smad4, and this complex translocates from the cytoplasm to the nucleus, where it associates with a variety of transcriptional coactivators (e.g., p300 [histone acetyltransferase p300] and CBP [CREB-binding protein]) or corepressors (e.g., TOB [transducer of ERBB2] and SIP1 [SMN-interacting protein 1]) to regulate gene transcription 17-20.

The Food and Drug Administration (FDA) in the United States has approved recombinant human BMP2 and 7 (rhBMP2 and 7) in the treatment of several bone-associated diseases, such as spinal fusions and nonunions, because these two BMPs can stimulate proteoglycan synthesis and promote bone formation 21,22. Although several studies have shown the promising benefits of rhBMPs to alleviate the IDD process, the underlying mechanisms are still obscure 23,24. In this report, we discovered the impairment of BMP/Smad signaling and the activation of Puma-dependent apoptotic signaling in the IDD specimens. Removal of BMPRI and BMPRII in NP cells could mimic the inhibition of BMP/Smad signaling and the induction of Puma-dependent apoptotic signaling. Importantly, we revealed that Smad4 assembled a transcriptional complex with HDAC1 (histone deacetylase 1) and the phosphorylated Smad1/5/8. This complex bound to the promoter of *PUMA* to repress its expression. Our results suggested that the decrease of BMPs impaired BMP/Smad signaling but activated Puma-dependent apoptotic signaling in IDD patients. We also provided evidence to support that supplementation of rhBMP2 and 7 could inhibit Puma-dependent apoptotic signaling *in vitro* and *in vivo*. Our findings suggest that the BMP/Smad signaling critically mediates Puma and its downstream events in the pathogenesis of IDD.

## Materials and methods

### Blood sample collection and enzyme-linked immunosorbent assay (ELISA)

Venous blood samples were collected from 20 healthy volunteers and 20 IDD patients who were under Pfirrmann grade IV. The IDD patients were all surgical treatment recipients in the Department of Orthopedics, Panzhihua Central Hospital between 2017 and 2019. The basic information of these participants is included in Supplementary Table 1. All participants gave informed consent that was approved by the Ethics Board of Panzhihua Central Hospital. The blood samples were immediately stored in blood collection tubes with K_2_EDTA (Thermo Fisher, Shanghai, China; #22-253-145). ELISA assays were performed to determine the circulating concentrations of BMPs using their individual kits, including BMP1 (Novus Biologicals, Centennial, CO, USA; #NBP2-69978), BMP2 (Abcam, Shanghai, China; #ab119581), BMP3 (Novus Biologicals; #NBP2-69993), BMP4 (Abcam; #ab231930), BMP5 (Abcam; #ab119583), BMP6 (Thermo Fisher; #EHBMP6), BMP7 (Abcam; #ab99985), BMP8A (CUSABIO, Wuhan, Hubei, China; #Q7Z5Y6), BMP8B (CUSABIO; #P34820), BMP10 (CUSABIO; #Q95393), and BMP15 (CUSABIO; #Q95972).

### Collection of IVD biopsies

The IVD tissues were collected from IDD patients under different Pfirrmann grades (I-IV, *n* = 1 for each grade) and one young patient (control) who had experienced a car accident and heavily damaged his IVD. The basic information of these participants was included in Supplementary Table 2. All participants gave informed consent that was approved by the Ethics Board of Panzhihua Central Hospital.

### Cell lines and transfection

The human NP cell line (HNPC) was purchased from ScienCell Research Laboratories (Carisbad, CA, USA; #4800). Cells were cultured in a NPCM (NP cell medium) (Carisbad; #4801) containing 10% fetal bovine serum (FBS) (Sigma-Aldrich, Shanghai, China; #F0926) and 100 U/mL penicillin-streptomycin (Sigma-Aldrich; #P4333). Cells were placed in a 37 °C incubator supplied with 5% CO_2_. For the cell transfection using shRNAs, two independent lentiviral transduction particles of each gene and the control particles were introduced into HNPC with lipofectamine 2000 (Thermo Fisher; #11668019) and hexadimethrine bromide (Sigma-Aldrich; #28728-55-4; final concentration: 8 µg/mL). The transfected cells were selected in NPCM containing 2 µg/mL puromycin for 10 days with medium change every 3 days. The puromycin-resistant colonies were individually collected and subjected to the required experiments. For the cell transfection using plasmids, the purified plasmids were transfected into HNPC with lipofectamine 2000. Cells were further incubated at 37 °C for another 48 hours and then subjected to the required experiments.

### Vector construction and plasmid purification

The full coding sequences of *PUMA*, *Smad4, BMPR1a,* and* BMPR2* were amplified using high-fidelity DNA polymerase (Thermo Fisher; #11304011) and then cloned into the pCDNA3-2×Flag empty vector. The full length of the coding sequence of *HDCA1* was cloned into the pCDNA3-Myc empty vector. The wild-type (WT) promoter (2,000 bp) of *PUMA* was amplified and cloned into the pGL4.26 luciferase empty vector. The obtained pGL4.26-PUMA^WT^ vector was used as a template to generate two-point mutations in which the Smad4-binding sites were mutated. Primers are listed in Supplementary Table 3.

### Total RNA extraction, microarray analysis, and real-time quantitative PCR (RT-qPCR) analysis

Cells (1×10^7^) under 80% confluence were washed with ice-cold PBS buffer (Sigma-Aldrich; #P5493) and then lysed using the TRI Reagent (Sigma-Aldrich; #93289) to isolate RNA. Total RNA was quantified using a spectrophotometer (Thermo Fisher; ND-2000), and cDNA was synthesized with the PrimeScript RT Reagent Kit (Takara, Beijing, China; RR037A). The microarray analysis was performed using the GeneChip Human Gene 2.0 ST Array (Thermo Fisher; #902112) following the guidelines of the manufacturer. RT-qPCR was performed using the Prelude PreAmp Mater Mix (Takara; #638541) with the primers listed in Supplementary Table-4. The PCR procedures included: 95 °C for 5 min, then 40 cycles of 95°C for 2 min, 68 °C for 30 sec, and finally 4 °C for 5 min. The relative gene expression levels were determined using the 2^-∆∆Ct^ method in which ∆∆Ct = Ct_(target gene)_-Ct_(β-actin)_.

### Western blotting

Cells (1×10^7^) under 80% confluence or IVD tissues (0.1 g) were lysed in 1 mL ice-cold radioimmunoprecipitation assay (RIPA) buffer (Thermo Fisher; #89901) containing the protease inhibitor (Abcam; #ab142778). Equal amounts (30 µg) of proteins were loaded into the wells of SDS-PAGE gel and separated by electrophoresis. Proteins were transferred onto the PVDF (polyvinylidene fluoride) membrane (Sigma-Aldrich; #IPSN07852) and blocked with 5% fat-free milk for one hour at room temperature. The membranes were then incubated with primary antibodies, including anti-BMPR1a (Abcam; #ab264043), anti-BMPR1b (Abcam; #ab175385), anti-BMPR2 (Abcam; #ab96826), anti-Smad1/5/8 (Sigma-Aldrich; #SAB2702532), anti-pSmad1/5/8 (Sigma-Aldrich; #AB3848-I), anti-Smad4 (Sigma-Aldrich; #HPA019154), anti-Puma (Abcam; #ab9645), anti-Apaf-1 (Abcam; #ab233786), anti-CASP9 (Abcam; #ab184786), anti-CASP3 (Thermo Fisher; #MA1-16843), anti-HDAC1 (Abcam; #ab7028), and anti-β-actin (Sigma-Aldrich; #A2066). After incubation at 4 °C overnight, membranes were washed 5 times with a PBS buffer containing 0.1% Tween-20 (Sigma-Aldrich; #P9416) and then probed with secondary antibodies (Abcam; #ab6721 and #ab6728). Protein signals were recorded by the Bio-Rad Gel Imaging System (Bio-Rad, Shanghai, China; #1708265).

### Immunoprecipitation (IP), mass spectrometry (MS), and co-IP assays

The degenerative IVD tissue (0.1 g) from an IDD patient under Pfirrmann grade IV was homogenized in 1 mL RIPA buffer containing the protease inhibitor. The supernatant of the cell lysate was immunoprecipitated using anti-Smad4- and IgG-coupled protein A beads (Santa Cruz Biotechnology, Shanghai, China; #sc-2001). The enriched proteins were rinsed five times with PBST buffer and then loaded onto a 12% SDS-PAGE gel for separation, followed by staining with the ProteoSilver Kit (Sigma-Aldrich; #PROTSIL2). The positive bands were cut into small pieces and then digested using the Trypsin Kit (Thermo Fisher Scientific; #60109101). The eluted proteins were subjected to MS analysis.

The co-IP assay was performed as described previously 25. In brief, different combinations of plasmids, including pCDNA3-2×Flag + pCDNA3-Myc, pCDNA3-2×Flag + pCDNA3-Myc-HDCA1, pCDNA3-2×Flag-Smad4 + pCDNA3-Myc-HDCA1, and pCDNA3-2×Flag-Smad4 + pCDNA3-Myc were co-transfected into HNPC cells. After 48 hours of transfection, cells were subjected to the IP procedure using anti-Flag agarose (Sigma-Aldrich; #A4596) and anti-Myc-agarose (Sigma-Aldrich; #A7470). The enriched protein complexes were probed with anti-Flag (Abcam; #ab125243) and anti-Myc (Abcam; #ab32).

### Luciferase assay

The pCL4.26-PUMA^WT^ and its mutant vectors were co-transfected with the pRL-Renilla luciferase control vector into Control-KD (knockdown), two Smad4-KD cell lines (#1 and #2), Control-OE (overexpression), and Smad4-OE cells. The resulting cells were cultured at 37 °C for another 24 hours and then subjected to a luciferase assay using the Pierce Renilla-Firefly Luciferase Dual Assay Kit (Thermo Fisher; #16186), following the manufacturer's guidelines.

### Chromatin immunoprecipitation (ChIP) assay

Cells (8×10^7^) were washed twice with ice-cold PBS buffer and then cross-linked using 1% formaldehyde (Creative Biolabs, Shirey, NY, USA; #Glyco-032CL) for 15 min at room temperature. The cross-linked DNA-protein complexes were sheared into ~500 bp DNA fragments by sonication, followed by ChIP assay with the Imprint ChIP Kit (Sigma-Aldrich; #CHP1-96RXN), following the manufacturer's protocol. The antibodies used for the ChIP assay included anti-Smad4, anti-Smad1, anti-HDAC1, and IgG (negative control). The resultant DNA was subjected to RT-qPCR analysis using the following primers: forward: 5'-ATCAGTATGTGAGTGTGTGTG-3' and reverse: 5'-GGTCCACAAAGTCACGTGCA-3'. Enrich fold was determined by the 2^-∆∆Ct^ method in which ∆∆Ct = Ct_(output)_-Ct_(input)_.

### Cell treatment

The HNPC cells under 80% confluence were exposed to 0.1 µM RITA (reactivating p53 and inducing tumor apoptosis) (Sigma-Aldrich; #506149) for three hours, followed by treatment with different concentrations (0, 10, and 20 ng/mL) of rhBMP2 (Sigma-Aldrich; #B3555) and rhBMP7 (Sigma-Aldrich; #SRP6157) for two hours. Cells were then washed twice with ice-cold PBS buffer, followed by RNA and protein isolation.

### Animal experiment

Sprague Dawley (SD) rats were purchased from the Cavens Company (Changzhou, Jiangsu, China). Eight-week-old rats (male, *n* = 15) with similar weights (200 ± 10 g) were randomly divided into four groups (*n* = 5 for each group): sham group, sham + PBS group, rhBMP2 group, and rhBMP7 group. The sham group was immediately subjected to magnetic resonance imaging (MRI) and X-rays to photograph the lumbar IVDs. The other three groups of rats were further grown in cages and intraperitoneally injected with PBS, 0.1 mg/kg rhBMP2, and 0.1 mg/kg rhBMP7 every 10 days, respectively. After administration of rhBMPs for one year, rats were subjected to MRI and X-rays to photograph the lumbar VDs. The IVD tissues in all groups were collected and used for protein isolation. The disc height index was measured and calculated following a previous protocol 26. The animal experiment was performed following a protocol approved by the Ethics Board of Panzhihua Central Hospital.

### Histologic analysis and immunohistochemistry (IHC) staining

Lumbar IVDs from different group of rats were fixed in 4% paraformaldehyde (Sigma-Aldrich; #158127). After dehydration, IVDs were embedded in paraffin, followed by cutting into 5-μm sections. The slices were then stained with hematoxylin-eosin (H&E) (Sigma-Aldrich; #1051750500) and images were photographed using a LEICA DM4000 B microscope. The IHC staining in NP tissues procedures were same as described previously 27. Antibodies were same as described in the western blot assay. The DAB (3,3′-Diaminobenzidine) kit was purchased from Abcam company (#ab64264).

### Statistical analysis

The microarray analysis was only performed once. All the other experiments were independently repeated in triplicate. Data were shown by the mean ± standard deviation (SD). The statistical significance was determined using a two-sided Student's *t*-test. *P* < 0.05 (*), *P*<0.01 (**) and *P*<0.001 (***).

## Results

### Circulating BMP levels were decreased and BMP/Smad signaling was impaired in IDD patients

**Figure 1:**
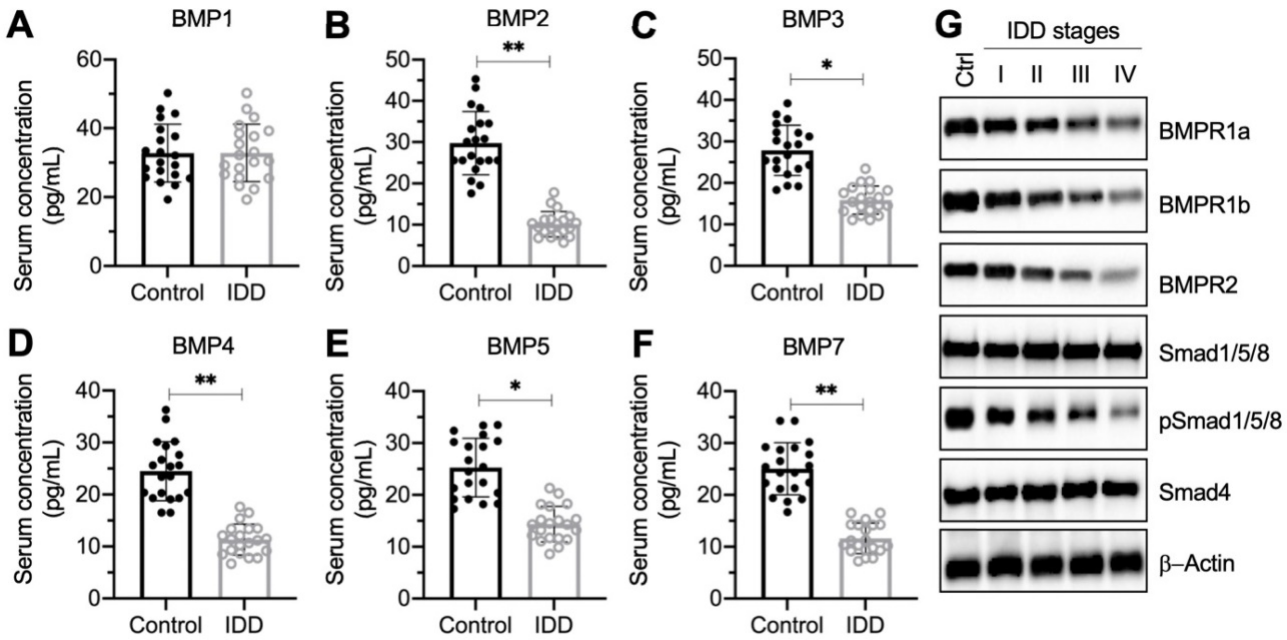
** The decrease of BMP circulating concentration and the deficiency of BMP/Smad signaling in IDD patients. (A-F)** Serum concentrations of BMPs. Circulating levels of BMP1 **(A)**, BMP2 **(B)**, BMP3 **(C)**, BMP4 **(D)**, BMP5 **(E)**, and BMP7 **(F)** were measured in serum samples obtained from healthy controls (*n* = 20) and IDD patients (*n* = 20). *P < 0.05 and **P < 0.01. **(G)** The protein levels of BMP/Smad signaling molecules. Total cell extracts from the IVDs in one control and different Pfirrmann grades (I-IV) were subjected to immunoblots to examine the protein levels of BMPR1a, BMPR1b, BMPR2, Smad1/5/8, pSSmad1/5/8, Smad4, and β-Actin (loading control).

To investigate whether circulating BMP levels were associated with IDD pathogenesis, we collected 20 pairs of blood samples from healthy controls and IDD patients under Pfirrmann grade IV. We measured the concentrations of all 11 BMPs (1, 2, 3, 4, 5, 6, 7, 8A, 8B, 10, and 15) in blood samples. The ELISA results showed that the serum concentrations of BMP1, BMP6, BMP8A, BMP8B, BMP10, and BMP15 were not significantly changed in IDD patients compared to controls (Figure 1A and Figure S1). The concentrations of the other five BMPs showed varying degrees of decline (Figure 1B-1F). In detail, the median concentrations for controls and IDD patients, respectively, were as follows: for BMP2, 32.1 ± 8.9 pg/mL compared with 9.5 ± 2.4 pg/mL (Figure 1B; *P* < 0.01); for BMP3, 28.7 ± 9.2 pg/mL compared with 14.3 ± 2.6 pg/mL (Figure 1C; *P* < 0.05); for BMP4, 24.2 ± 6.0 pg/mL compared with 12.5 ± 2.7 pg/mL (Figure 1D; *P* < 0.01); for BMP5, 24.5 ± 7.1 pg/mL compared with 13.6 ± 3.3 pg/mL (Figure 1E; *P* < 0.05); and for BMP7, 24.4 ± 5.9 pg/mL compared with 10.1 ± 3.1 pg/mL (Figure 1F; *P* < 0.01).

In addition, we also collected IVD tissues from one nondegenerative control and from IDD patients under different Pfirrmann grades (from I-IV, *n* = 1 for each grade). The immunoblot results indicated that the protein levels of Smad1/5/8 and Smad4 were not changed in differently sourced IVD tissues (Figure 1G and Figure S2). However, two BMP receptors and the phosphorylated Smad1/5/8 were gradually decreased following the severity of IVD degeneration (Figure 1G and Figure S2). These results suggested that the BMP/Smad signaling was disrupted in IDD specimens.

### *In vitro* knockdown of *Smad4* significantly induced the expression of *PUMA*

**Figure 2:**
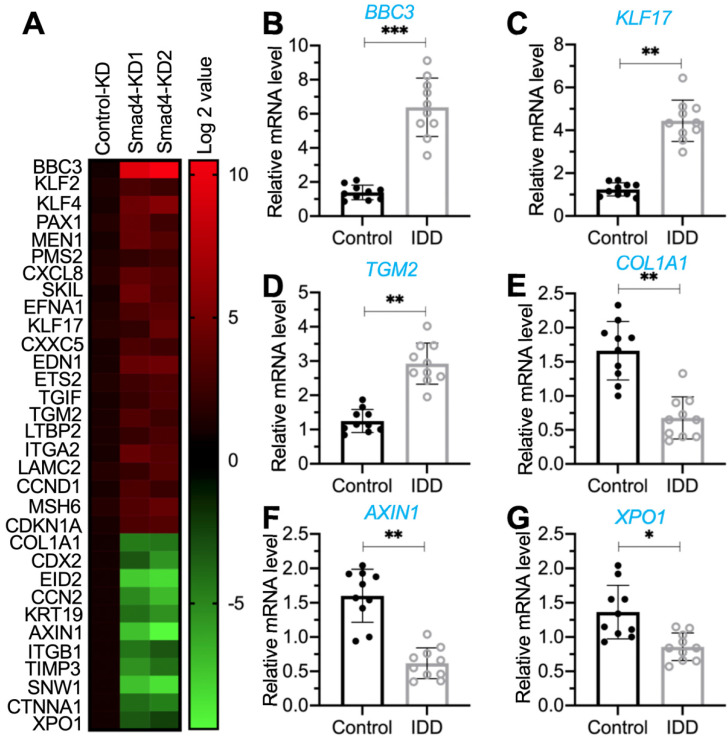
** Identification of aberrantly expressed genes dependent on Smad4 and verification of their expression levels in the blood leukocyte samples from IDD patients. (A)** The heat map of Smad4-dependent genes. Total RNA samples from Control-KD, Smad4-KD1, and Smad4-KD2 cells were used for microarray analysis. The aberrantly expressed genes are shown. The color red or green reflected relatively high or low expression levels by normalizing to control, respectively, which was indicated in the scale bar (log2-transformed scale). (B-G) Detection of gene expression levels in the blood leukocyte samples from IDD patients. Total RNA samples isolated from leukocyte samples in control and IDD patients were subjected to RT-qPCR analyses to detect the expression levels of six genes: *PUMA* **(B)**, *KLF17* **(C)**, *TGM2* **(D)**, *COL1A1* **(E)**, *AXIN1* **(F)**, and *XPO1* **(G)**. *P < 0.05, **P < 0.01, and ***P < 0.001.

The translocation of the pSmad1/5/8-Smad4 complex from the cytoplasm to the nucleus is required for the transcription of genes 17-20. The impairment of BMP/Smad signaling in IDD patients inspired us to investigate the downstream targets of the pSmad1/5/8-Smad4 complex and reveal their roles in the pathogenesis of IDD. For this purpose, we generated two independent Smad4-KD cell lines in the HNPC background (Figure S3). Using the Control-KD and Smad4-KD cells, we performed a microarray analysis to identify Smad4-dependent genes. In total, we identified 32 dysregulated genes (21 upregulated and 11 downregulated genes) that were consistent in two Smad4-KD cell lines (Figure 2A and Supplementary Table 5). To determine the expression levels of these dysregulated genes in IDD patients, we collected blood leukocyte samples from 20 healthy controls and 20 IDD patients under Pfirrmann grade IV and then measured the mRNA levels of *PUMA*, *KLF17* (Krüppel-like factor 17), *TGM2* (transglutaminase 2), *COL1A1* (collagen type I alpha 1 chain), *AXIN1* (axin 1), and *XPO1* (exportin 1) as examples. The RT-qPCR analyses showed that *PUMA*, *KLF17*, and *TGM2* mRNA levels were significantly increased in IDD-sourced leukocyte samples (Figures 2B-2D). In contrast, the expression levels of *COL1A1*, *AXIN1*, and *XPO1* were downregulated in the same samples (Figures 2E-2G).

After analyzing these dysregulated genes, we identified that *PUMA* was mostly upregulated (increasing approximately six-fold) following the repression of *Smad4* (Figure 2B and Supplementary Table 5). Given that Puma is a proapoptotic protein and the activation of apoptosis is one of the major mechanisms that cause IDD pathogenesis, we will focus our study on revealing whether Smad4 and its associated transcription complex can regulate the expression of *PUMA* in the following experiments.

### Puma-dependent apoptotic signaling was activated in IDD patients, and rhBMPs could block Puma-dependent apoptotic signaling *in vitro*

**Figure 3:**
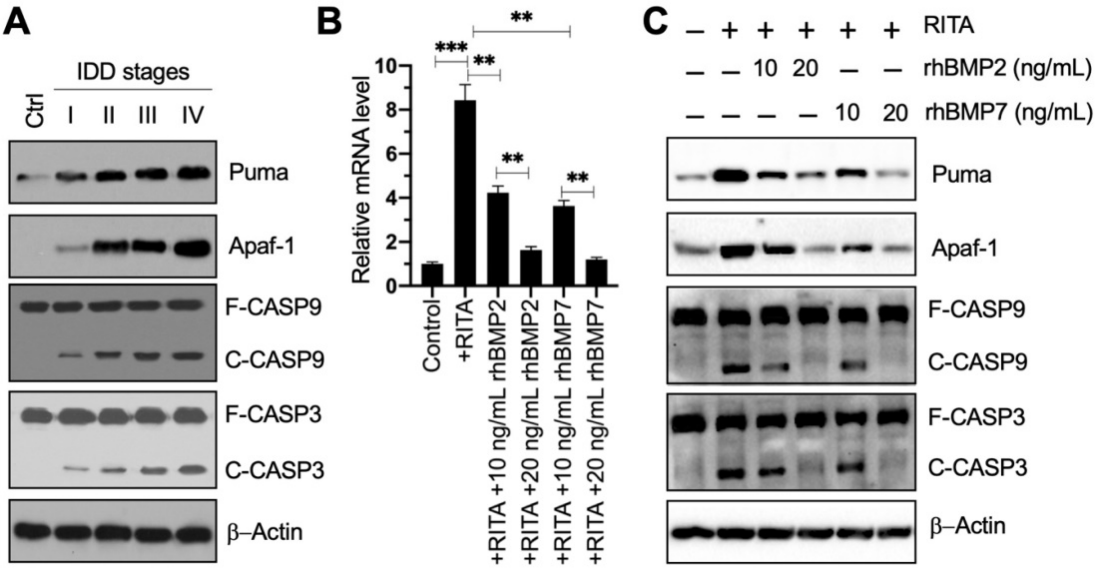
** Puma and its downstream apoptotic signaling were activated in IDD patients, and rhBMPs could block the activation of PUMA-dependent apoptotic signaling. (A)** Protein levels of Puma and its downstream apoptotic molecules in IDD specimens. Total cell extracts from the IVDs in one control and different Pfirrmann grades (I-IV) were subjected to immunoblots to examine the protein levels of Puma, Apaf-1, CASP9, CAPS3, and β-Actin (loading control). F: full length; C: cleaved. **(B)** *PUMA* mRNA level. The HNPC cells were treated with 0.1 µM RITA for three hours, followed by incubating with different concentrations (0, 10, and 20 ng/mL) of rhBMP2 and rhBMP7 for two hours. Total RNA samples were subjected to RT-qPCR analysis to examine the PUMA mRNA level. **P < 0.01 and ***P < 0.001. **(C)** Protein levels of Puma and its downstream apoptotic molecules in RITA- and rhBMP-treated cells. Total cell extracts from cells used in (B) were subjected to immunoblots to examine the protein levels of Puma, Apaf-1, CASP9, CAPS3, and β-Actin (loading control).

The result that *PUMA* was upregulated in blood leukocyte samples inspired us to investigate the expression level of its encoding protein Puma in IDD specimens. Using the control and degenerative IVD samples, we performed immunoblots to examine the protein levels of Puma and its downstream apoptotic molecules. Our results showed that Puma gradually accumulated following the increase of Pfirrmann grades (Figure 3A and Figure S4A). Similarly, the apoptotic molecules, including Apaf-1, Caspase-9, and Caspase-3, were also activated and showed similar patterns to that of Puma in IDD specimens (Figure 3A and Figure S4A). These results suggested that Puma-dependent apoptotic signaling was activated in IDD patients.

Since Smad4 can negatively regulate the expression of *PUMA*, we next sought to determine if the activation of BMP/Smad signaling could change the expression of *PUMA*. For this purpose, we treated HNPC cells using RITA to activate p53-dependent apoptotic signaling and then exposed the cells to two doses of rhBMP2 and rhBMP7. As expected, we found that RITA significantly induced the expression of *PUMA*, while rhBMPs dose-dependently reversed the expression of *PUMA* induced by RITA (Figure 3B). Moreover, we also examined the protein levels of Puma and its downstream apoptotic molecules. The protein levels of Puma, Apaf-1, cleaved Caspase-9, and cleaved Caspase-3 were significantly increased following RITA treatment, and they could be dose-dependently repressed by the treatments of rhBMPs (Figure 3C and Figure S4B). These results suggested that rhBMPs could block Puma-dependent apoptotic signaling *in vitro*.

### Removal of BMP receptors in NP cells activated Puma-dependent apoptotic signaling

**Figure 4:**
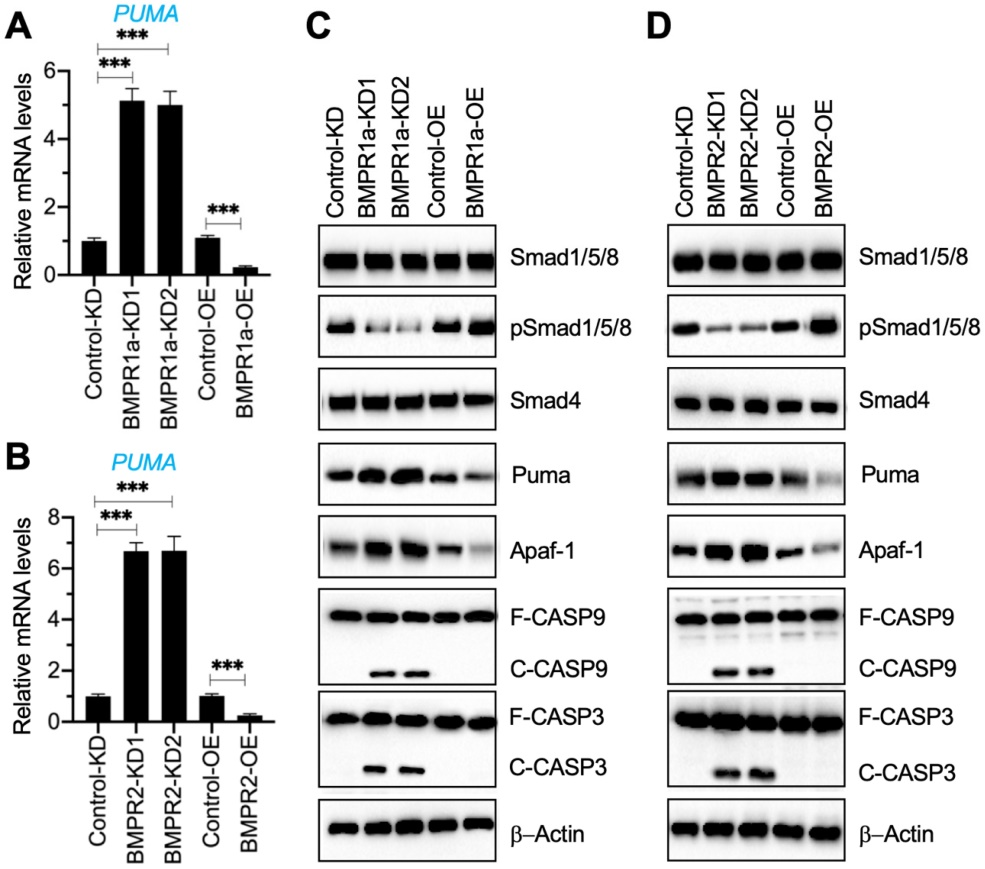
** Downregulation of BMPRs activated Puma and its downstream apoptotic molecules. (A)** *PUMA* mRNA level in BMPR1a-KD and BMPR1a-OE cells. Total RNA samples from Control-KD, BMPR1a-KD1, BMPR1a-KD2, Control-OE, and BMPR1a-OE cells were used for RT-qPCR analysis to examine the mRNA level of *PUMA*. ***P < 0.001. **(B)** *PUMA* mRNA level in BMPR2-KD and BMPR2-OE cells. Total RNA samples from Control-KD, BMPR2-KD1, BMPR2-KD2, Control-OE, and BMPR2-OE cells were used for RT-qPCR analysis to examine the mRNA level of *PUMA*. ***P < 0.001. **(C)** The protein levels of BMP/Smad signaling molecules and Puma-dependent apoptotic molecules in BMPR1a-KD and BMPR1a-OE cells. Total cell extracts from cells used in (A) were subjected to examine protein levels, as shown in the figure. **(D)** The protein levels of BMP/Smad signaling molecules and Puma-dependent apoptotic molecules in BMPR2-KD and BMPR2-OE cells. Total cell extracts from cells used in (B) were subjected to examined protein levels, as shown in the figure.

Our results in Figure 1G and Figure 3A indicated a decrease in BMP receptors but an increase in Puma-dependent apoptotic molecules in degenerative IVDs. To further determine if the activation of Puma-dependent apoptotic signaling was caused by the impairment of BMP/Smad signaling, we generated two independent BMPR1a-KD, one BMPR1a-OE, two independent BMPR2-KD, and one BMPR2-OE cell line in the HNPC background (Figure S5). Using these cells, we measured the mRNA level of *PUMA* and protein levels of BMP/Smad and Puma downstream apoptotic molecules. As shown in Figures 4A and 4B, the knockdown of *BMPR1a* and *BMPR2* caused a dramatic increase in the* PUMA* mRNA level, while their overexpression resulted in the downregulation of *PUMA*. The immunoblot results indicated that knockdown of *BMPR1a* and *BMPR2* caused the downregulation of pSmad1/5/8 but the accumulation of Puma, Apaf-1, cleaved Caspase-9, and cleaved Caspase-3 (Figures 4C, 4D, and Figure S6). In contrast, overexpression of *BMPR1a* and *BMPR2* increased the phosphorylation of Smad1/5/8 and decreased the protein levels of Puma, Apaf-1, cleaved Caspase-9, and cleaved Caspase-3 (Figures 4C, 4D, and Figure S6). The results suggested that the removal of BMP receptors *in vitro* impaired the BMP/Smad signaling and activated Puma-dependent apoptotic signaling.

### Smad4 bound to the promoter of *PUMA* to negatively regulate its expression

**Figure 5:**
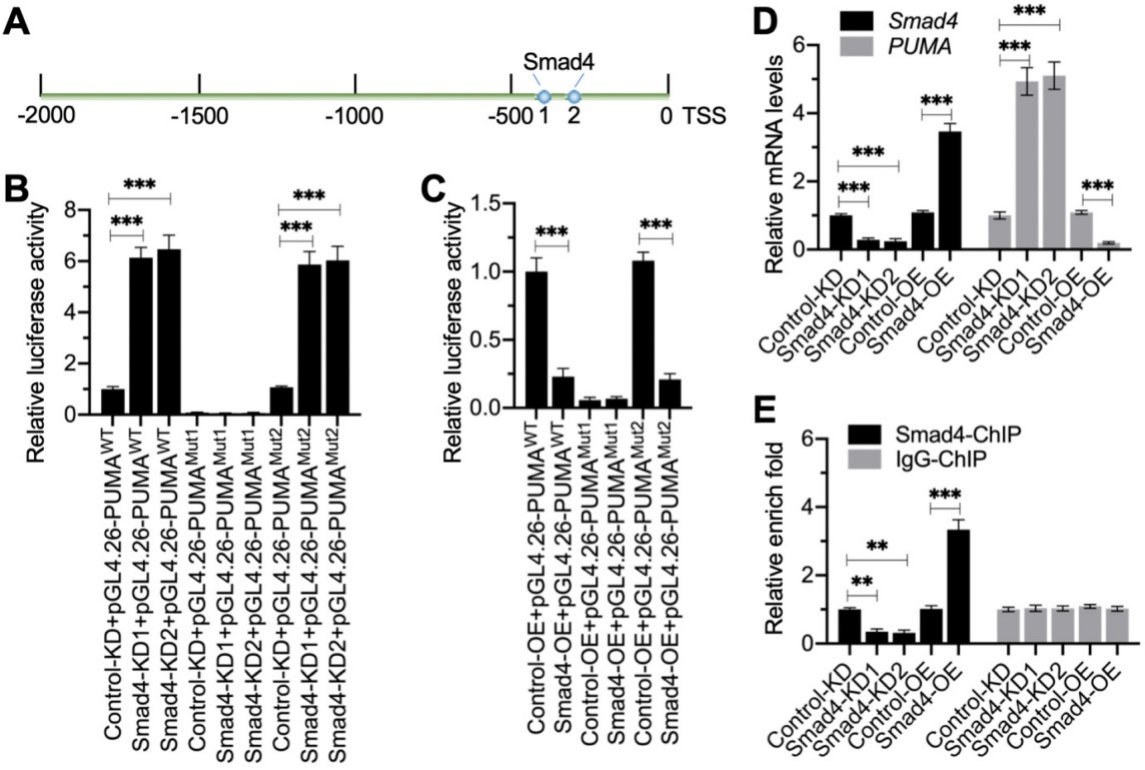
** Smad4 docked onto the promoter of *PUMA* at Site 1 to negatively regulate expression of *PUMA.* (A)** The promoter of *PUMA* contained two putative Smad4 binding sites. A 2,000-bp length of the *PUMA* promoter was used to identify the binding sites of Smad4. Two binding sites (1 and 2) were identified, and their positions were shown. **(B)** Relative luciferase activities in Smad4-KD cells. Three plasmids, pGL4.26-PUMA^WT^, pGL4.26-PUMA^Mut1^, and pGL4.26-PUMA^Mut2^, were co-transfected with Renilla into Control-KD, Smad4-KD1, and Smad4-KD2 cells. The relative luciferase activities were determined using a dual-luciferase reporter assay by normalizing the firefly luciferase activities to their corresponding Renilla activities. ***P < 0.001. **(C)** Relative luciferase activities in Smad4-OE cells. The plasmids used in (B) were co-transfected with Renilla into Control-OE and Smad4-OE cells. The relative luciferase activities were determined by normalizing the firefly luciferase activities to their corresponding Renilla activities. ***P < 0.001. **(D)** *Smad4* and *PUMA* mRNA levels. Total RNA samples from Control-KD, Smad4-KD1, Smad4-KD2, Control-OE, and Smad4-OE cells were subjected to RT-qPCR analyses to examine the mRNA levels of *Smad4* and *PUMA*. ***P < 0.001. **(E)** The occupancy of Smad4 on the promoter of *PUMA*. Cells used in (D) were subjected to ChIP assays using anti-Smad4 and IgG (negative control). The input and output DNA samples were subjected to RT-qPCR analysis to examine the occupancy of Smad4 on the *PUMA* promoter. **P < 0.01 and *** *P* < 0.001.

We next aimed to investigate whether Smad4 could bind to the promoter of *PUMA*. First, we analyzed the promoter of *PUMA* (2,000 bp) to identify the Smad4 binding sites. Blasting with the consensus sequence of GGCGCCN_5_GTCT, we identified two potential Smad4 binding sites (-339-[-353] and -407-[-420]) on the promoter of *PUMA* (Figure 5A). We labeled the -407-(-420) site as Site 1 (GTGGCCTTGTGTCT) and the -339-(-353) site as Site 2 (CCCGTCGGTCGGTCT) (Figure 5A). To determine which site was essential for the binding of Smad4, we created the WT and mutated promoters (changing GTCT to AGAC) in the pGL4.26 luciferase vector and then individually transfected these vectors with Renilla into Control-KD, Smad4-KD (#1 and #2), Control-OE, and Smad4-OE cells (Figure S7). The luciferase assay results indicated that knockdown of *Smad4* resulted in the significant induction of luciferase activities when cells were transfected into vectors containing WT and Mutant 2 (Site 2 mutation) (Figure 5B). The mutation of Site 1 failed to induce luciferase activities in Smad4-KD cells compared to Control-KD cells (Figure 5B). In contrast, overexpression of Smad4 significantly decreased luciferase activities in cells expressing pGL4.26-PUMA^WT^ and pGL4.26-PUMA^Mutant2^ but not in cells expressing pGL4.26-PUMA^Mutant1^ (Figure 5C). These results suggested that Site 1 was required for the binding of Smad4.

To further solidify the conclusion that Smad4 negatively regulated the expression of *PUMA*, we next examined the mRNA level of *PUMA* in Smad4-KD and Smad4-OE cells. The RT-qPCR results showed that *PUMA* was upregulated in Smad4-KD cells, while it was downregulated in Smad4-OE cells (Figure 5D). We also performed ChIP assays using anti-Smad4 and IgG-coupled protein A agarose. The ChIP results indicated that the binding of Smad4 to the promoter of *PUMA* was significantly decreased in Smad4-KD cells but increased in Smad4-OE cells (Figure 5E).

### The pSmad1/5/8-DCAF1-Smad4 transcriptional complex docked on the promoter of *PUMA* to negatively regulate its expression

**Figure 6:**
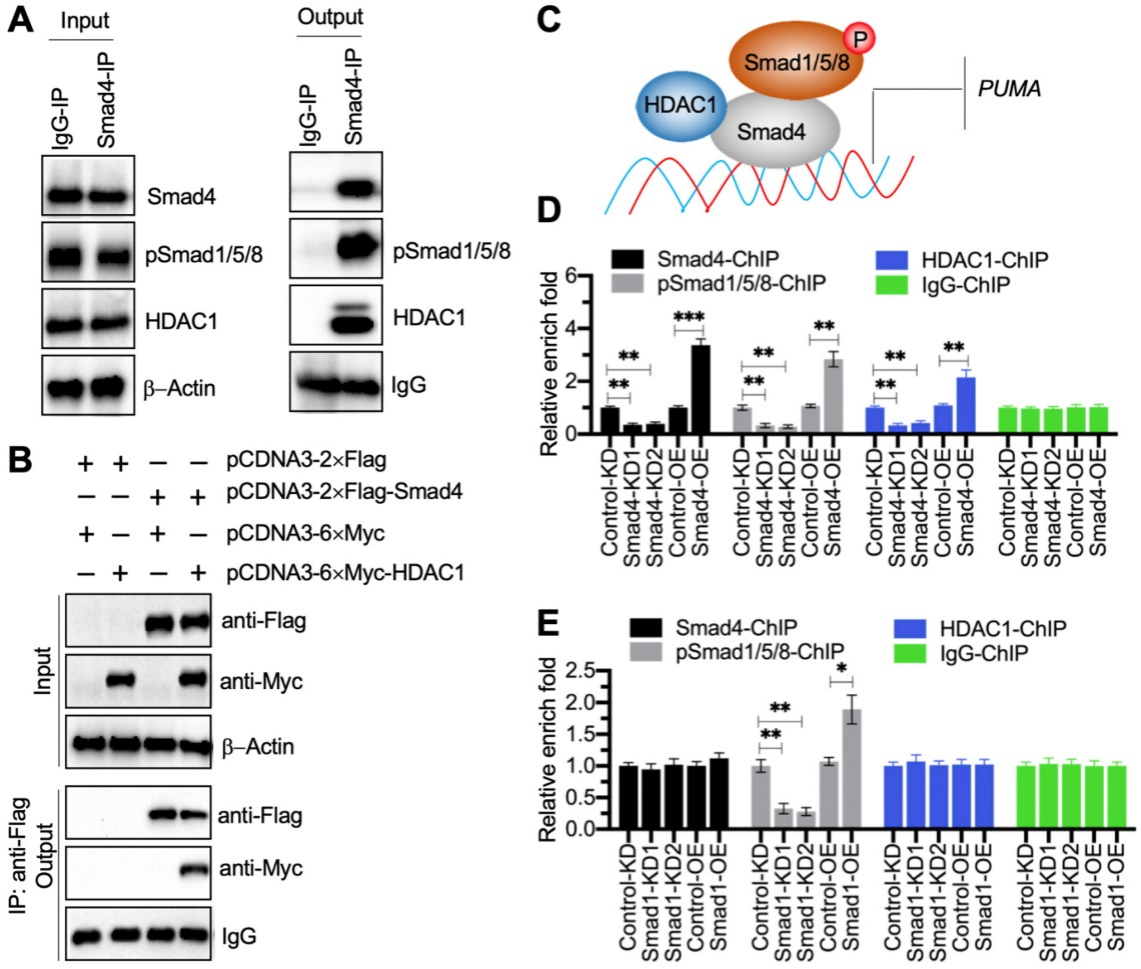
** Smad4 recruited HDAC1 and pSmad1/5/8 to assemble a transcriptional complex. (A)** Smad4 could pull down pSmad1/5/8 and HDAC1 *in vivo*. A degenerative IVD from an IDD patient under Pfirrmann grade IV was subjected to IP assays with IgG and anti-Smad4. The input and output proteins were used to examine the protein levels of Smad4, pSmad1/5/8, and HDAC1. **(B)** HDAC1 directly interacted with Smad4 *in vitro*. Cells expressing different plasmids shown in the figure were subjected to co-IP assays with Flag-agarose and Myc-agarose. The input and output proteins were subjected to immunoblots using anti-Flag and anti-Myc antibodies. **(C)** A schematic diagram of the pSmad1/5/8-HDAC1-Smad4 complex. **(D)** ChIP results in Smad4-KD and Smad4-OE cells. The Control-KD, Smad4-KD1, Smad4-KD2, Control-OE, and Samd4-OE cells were subjected to ChIP assays using anti-Smad4, anti-pSmad1/5/8, anti-HDAC1, and IgG, respectively. The input and output DNA samples were subjected to RT-qPCR analysis. **P < 0.01 and ***P < 0.001. **(E)** ChIP results in Smad1-KD and Smad1-OE cells. The Control-KD, Smad1-KD1, Smad1-KD2, Control-OE, and Samd1-OE cells were subjected to ChIP assays using anti-Smad4, anti-pSmad1/5/8, anti-HDAC1, and IgG, respectively. The input and output DNA samples were subjected to RT-qPCR analysis. *P < 0.05 and **P < 0.01.

Transcription factors often cooperate with other transcriptional regulators and proteins to control gene expression 28. To determine the Smad4-associated transcriptional complex components, we performed an IP assay using degenerative IVD tissue derived from a patient with Pfirrmann grade IV. Except for the pSmad1/5/8, we discovered a transcriptional regulator HDAC1 in the candidate proteins associated with Smad4 (Supplementary Table 6). Using the *in vivo* IP products, we verified that Smad4 could pull down both HDAC1 and pSmad1/5/8 (Figure 6A). We also performed an *in vitro* co-IP assay to verify that HDAC1 could directly interact with Smad4 (Figure 6B). Thus, we speculated that HDAC1 and pSmad1/5/8 could interact with Smad4 at different positions to assemble the pSmad1/5/8-DCAF1-Smad4 transcriptional complex (Figure 6C). To verify the accuracy of this model, we performed ChIP assays using anti-Smad4, anti-HDAC1, and anti-pSmad1/5/8 in Smad4-KD, Smad4-OE, Smad1-KD, Smad1-OE, HDAC1-KD, and HDAC1-OE cells, respectively. The expression level of *HDAC1* in its KD and OE cells were shown in Figure S8A. The ChIP results in Smad4-KD and Smad4-OE cells indicated that the occupancies of Smad4, HDAC1, and pSmad1/5/8 were all significantly decreased in Smad4-KD cells but increased in Smad4-OE cells (Figure 6D). Interestingly, we found that the occupancies of Smad4 and HDCA1 did not change in Smad1-KD and Smad1-OE cells, while the enrichment of pSmad1/5/8 was decreased in Smad1-KD cells but increased in Smad1-OE cells (Figure 6E). Similarly, the occupancies of Smad4 and pSmad1/5/8 did not change in HDCA1-KD and HDAC1-OE cells, while the enrichment of HDCA1 was decreased in HDCA1-KD cells but increased in HDAC1-OE cells (Figure S8B). In addition, we also measured *PUMA* mRNA level in HDAC1-KD and HDAC1-OE cells. The RT-qPCR results showed that *PUMA* was increased in HDAC1-KD cells but decreased in HDAC1-OE cells (Figure S8C). These results supported our model in which Smad4 docked on the promoter of *PUMA*, and Smad4 then recruited HDCA1 and pSmad1/5/8 to assemble a complex.

### Administration of rhBMPs suppressed the degeneration of IVDs in rats

**Figure 7:**
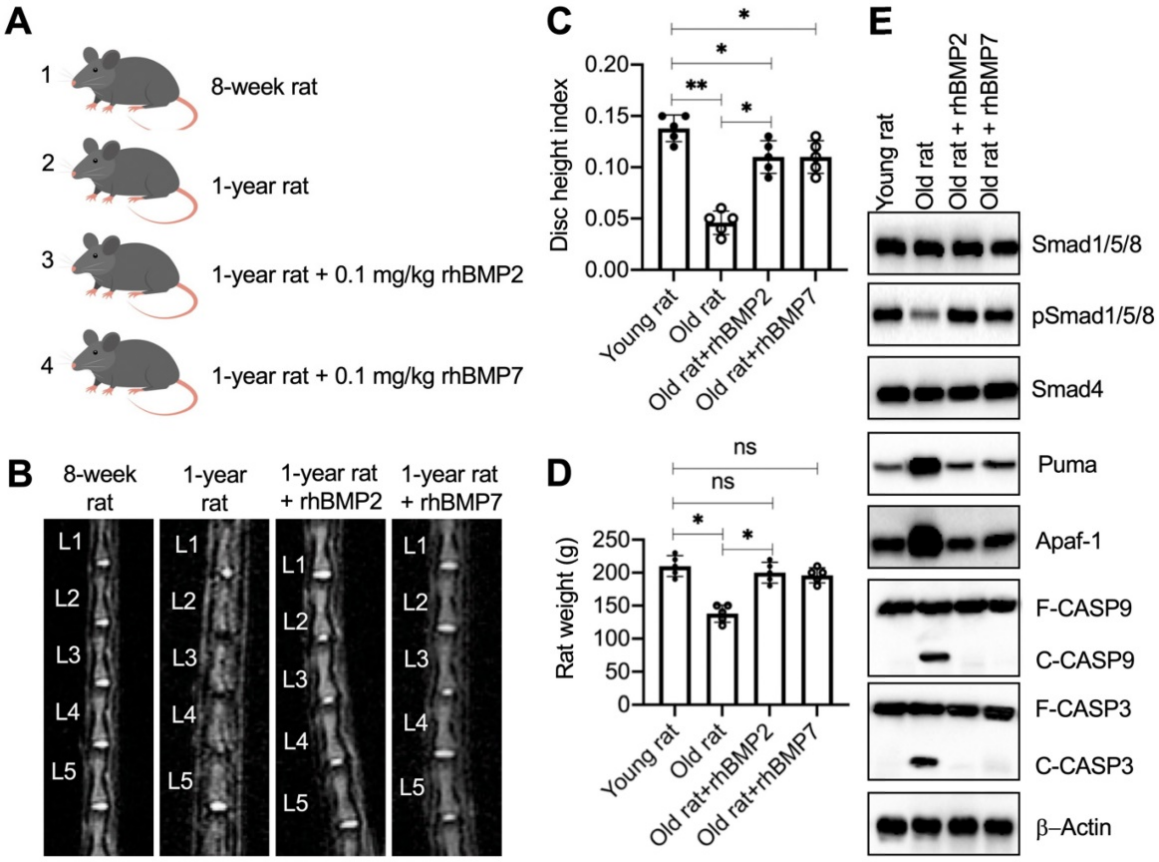
** rhBMPs inhibited the degeneration of IVDs in aged rats. (A)** A schematic diagram of different groups of rats. **(B)** MRI images of lumbar IVDs. Different groups of rats (*n* = 5 for each group) were used for MRI images, and the representative lumbar IVDs (L1-L5) were shown. **(C)** Disc height index (DHI). The DHI was calculated based on lumbar vertebrae. *P < 0.05 and **P < 0.01. **(D)** Weights of rats. Different groups (*n* = 5 for each group) of rats were weighed at the end of the experiments. ns=no significant difference. *P < 0.05. **(E)** The protein levels of BMP/Smad signaling molecules and Puma-dependent apoptotic molecules in rats. Total cell extracts from lumber IVDs collected from different groups of rats were subjected to examination of protein levels, as shown in the figure.

The significant improvement of rhBMPs in blocking Puma-dependent apoptotic signaling *in vitro* inspired us to evaluate their *in vivo* effects. For this purpose, we randomly divided rats into four groups: sham group, sham + PBS group, rhBMP2 group, and rhBMP7 group. The young rats in the sham group were immediately subjected to MRI and X-rays to image the lumbar IVDs and collected IVDs. The other three groups of rats were further grown in cages and intraperitoneally injected with PBS, 0.1 mg/kg rhBMP2, and 0.1 mg/kg rhBMP7 every 10 days, respectively (Figure 7A). After administration of rhBMPs for one year, rats were subjected to MRI and X-rays to image the lumbar IVDs and collected IVDs. The MRI and X-ray results showed that the lumbar IVDs in rats injected with PBS were significantly degenerated in comparison to the young rats (Figures 7B, 7C, S9A, and S9B). However, the administration of rhBMPs significantly suppressed the degeneration of lumbar IVDs (Figures 7B, 7C, S9A, and S9B). The degenerative changes of lumbar IVDs in different groups of rats were also observed using histological assay (Figure S9C). In addition, we also measured the weights of rats in different groups and found that the average weights in the sham + PBS group of rats were obviously decreased when they became old (Figure 7D). The weights in rhBMP-administrated rats were similar to those in the young group (Figure 7D).

We also examined the molecular changes of the BMP/Smad and Puma downstream apoptotic proteins. As shown in Figure 7E and S10A, we observed the downregulation of pSmad1/5/8 but the accumulation of Puma, Apaf-1, cleaved Caspase-9, and cleaved Caspase-3 in the sham + PBS group of rats. These changes were restored by the administration of rhBMPs (Figure 7E and S10A). In addition, we also performed IHC assays to detect the expression changes of Smad4, Puma, and Apaf-1 in lumbar IVDs from different groups of rats. Consistent with the western blot results, we also observed the similar changes of Puma and Apaf-1 in different groups of rats (Figure S10B). The results suggested that Puma-dependent apoptotic signaling could be blocked by rhBMPs *in vivo*.

## Discussion

**Figure 8:**
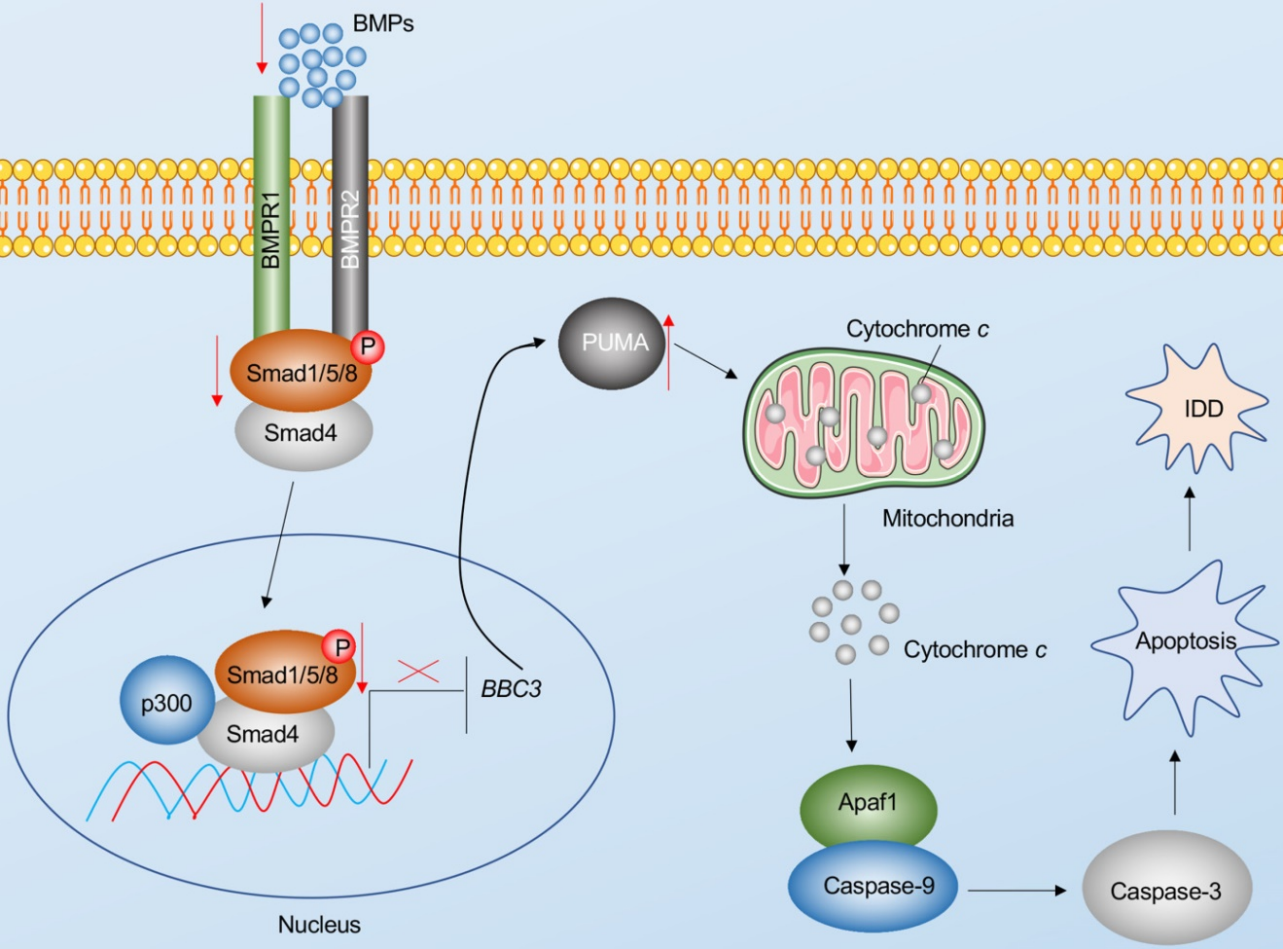
** Schematic diagram of BMP-mediated activation of Puma-dependent apoptotic signaling in the pathogenesis of IDD.** The decreased levels of BMPs failed to activate the BMPR1 and BMPR2 receptors on the membrane, causing the decrease of phosphorylation of Smad1/5/8 and the failed assembly of the pSmad1/5/8-Smad4 complex. The failed translocation of the pSmad1/5/8-Smad4 complex from the cytoplasm to the nucleus limits its control in the transcription of *PUMA*, resulting in the upregulation of *PUMA*. Puma induces the release of cytochrome *c* from the mitochondria. Cytochrome *c* binds to Apaf-1, which recruits caspase-9 to assemble the apoptosome, leading to the activation of Caspase-9 and Caspase-3. The activation of apoptosis causes the occurrence of IDD.

Apoptosis is a major basis for the pathogenesis of IDD 7. In the present study, we reveal Puma-dependent apoptotic signaling that is initiated by the decrease of BMPs in the pathogenesis of IDD. The decrease of BMPs fails to activate their receptors on the cell membrane, decreasing the phosphorylation of Smad1/5/8 and impairing the assembly of the pSmad1/5/8-HDAC1-Smad4 transcriptional complex. This complex negatively regulates the expression of *PUMA* by docking on its promoter. The impairment of the pSmad1/5/8-HDAC1-Smad4 complex causes the induction of *PUMA* and the accumulation of Puma, activating the Puma downstream events, including the release of cytochrome *c* from the mitochondria and the activation of Apaf-1, Caspase-9, and Caspase-3, eventually leading to the pathogenesis of IDD (Figure 8).

The BMP/Smad signaling is an ancient and highly conserved pathway in mammals 29. The BMP superfamily members affect most biological processes of bone and cartilage 29. Dysregulated BMP/Smad signaling has been observed in many human skeletal disorders, such as nonunion, Loeys-Dietz syndrome, and orofacial cleft 29,30. Modulation of rhBMP2, rhBMP4, and rhBMP7 has been developed as a therapeutic strategy to stimulate osteogenesis, improve bone mass and quality, and repair damaged bones and joints 29,30. Although these rhBMPs have been widely used in spinal surgery for nearly two decades, their molecular effects are still being investigated. Importantly, the roles and downstream targets of BMP/Smad signaling are still obscure in the pathogenesis of IDD. Recently, a study has also reported that rhBMP2 can alleviate the IDD process in a rat model by mediating the degradation of the extracellular matrix and inhibiting apoptosis via the PI3K/PKB (phosphatidylinositol 3-kinase/protein kinase B) pathway 31. Our results in human blood samples and IVD tissues suggest that BMP/Smad signaling is deficient in IDD patients. Although we found 32 potential targets of Smad4, we only focused our study on *PUMA* due to the important role of apoptosis in the pathogenesis of IDD. The pathogenesis of IDD may be caused by a variety of genes and signaling pathways. Thus, more efforts are required to investigate the contributions of the other 31 candidate genes in the future. The current study identifies a link between the BMP/Smad signaling and Puma-dependent apoptotic signaling, which provides an avenue for investigating the underlying mechanisms of other skeletal disorders.

Puma is a critical mediator of apoptotic signaling and can be induced by different stimuli, such as genotoxic stress, redox microenvironment, deficient cytokines, and infection 11-13. In addition, *PUMA* can be transcriptionally regulated by different transcription factors, such as p53, c-Myc, and FOXO3a (forkhead box O3a) 32. In this study, we identified a new transcriptional mechanism mediated by the pSmad1/5/8-HDAC1-Smad4 complex. The assembly of this transcriptional complex is controlled by BMPs, and the deficiency of BMPs in IDD patients caused the impaired assembly of pSmad1/5/8-Smad4, thus decreasing the transcriptional efficiency of the whole pSmad1/5/8-HDAC1-Smad4 complex. HDAC1 is a histone deacetylase and plays a key role in the suppression of gene expression by interacting with transcription factors and causing the deacetylation of transcription factors 33,34 Previous studies have shown that Smad4 can recruit transcriptional coactivator p300/CBP to activate gene expression 35,36. Our current study identifies a new transcriptional complex in which Smad4 recruits HDAC1 to repress the expression of *PUMA*, which enriches the regulatory mechanisms of Smad4 and suggests that Smad4 may recruit different transcriptional regulators to control gene expression.

In summary, we found that lower levels of BMPs caused the deficiency of BMP/Smad signaling in IDD patients, leading to the impairment of the pSmad1/5/8-HDAC1-Smad4 complex and resulting in the induction of *PUMA*. The accumulation of the *PUMA*-encoding protein PUMA initiated apoptosis and resulted in the occurrence of IDD. The administration of rhBMP2 and 7 significantly inhibit IDD process by blocking the Puma-dependent apoptotic signaling.

## Supplementary Material

Supplementary figures and tables.
