# Supplementary material for: Recombinant human bone morphogenetic protein 2 and 7 inhibit the degeneration of intervertebral discs by blocking the Puma-dependent apoptotic signaling
Source: Int J Biol Sci. 2021 Jun 11;17(9):2367–79. doi: 10.7150/ijbs.56823 (PMC8241732; doi:10.7150/ijbs.56823)
Supplement: Supplementary file 1 — Supplementary figures and tables. [file ijbsv17p2367s1.pdf]

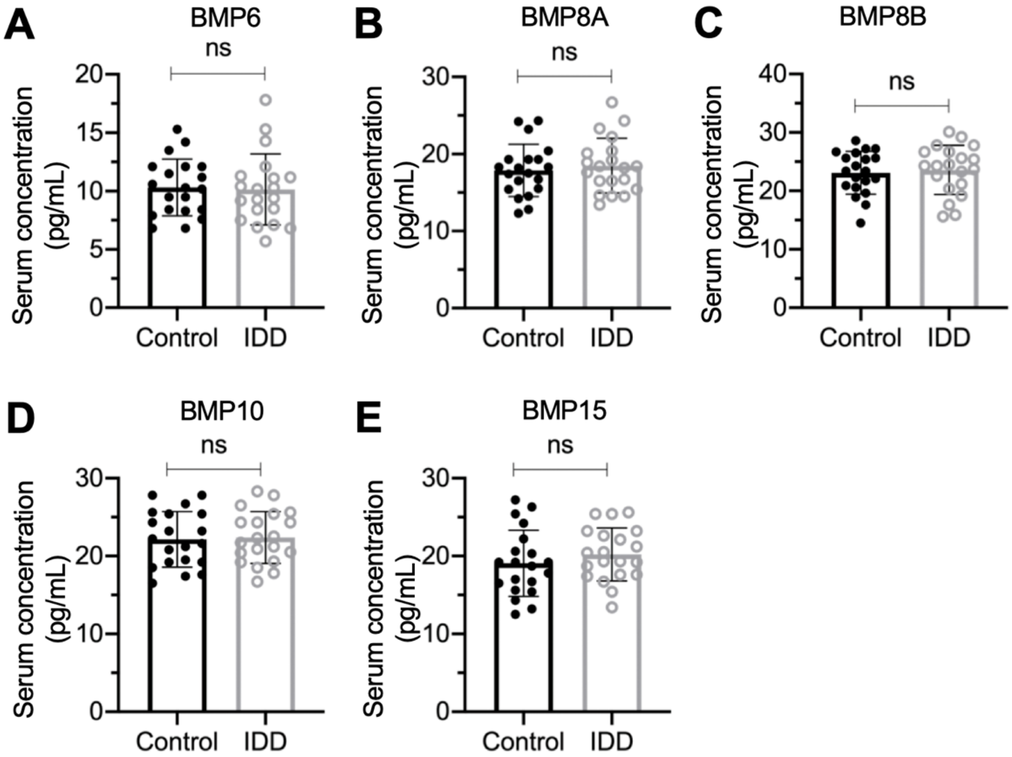

Figure S1. The circulating concentrations of BMPs in IDD patients

Circulating levels of BMP6 **(A)**, BMP8A **(B)**, BMP8B **(C)**, BMP10 **(D)**, and BMP15 **(E)**, were measured in serum samples obtained from healthy controls (*n* = 20) and IDD patients (*n* = 20) by ELISA assays. ns represents no significant difference.

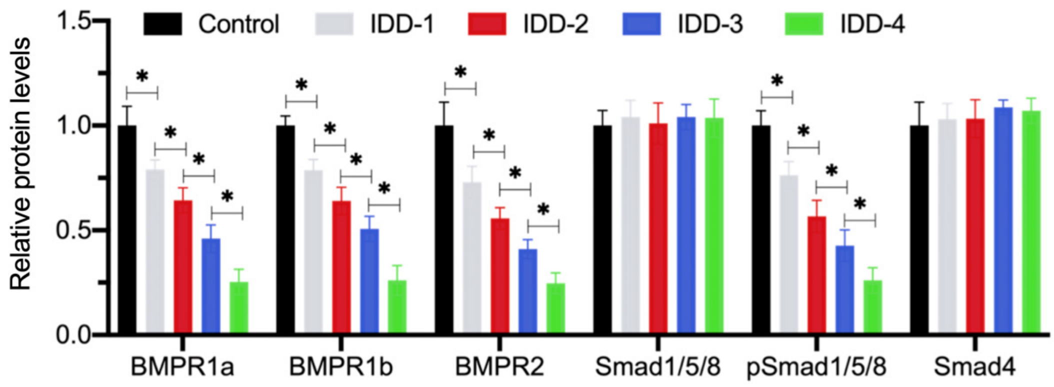

Figure S2. The relative protein levels of BMP/Smad signaling molecules in IDD patients

The protein bands in Figure 1G were quantified using Image J software and then normalized to their corresponding loading controls. *P < 0.05.

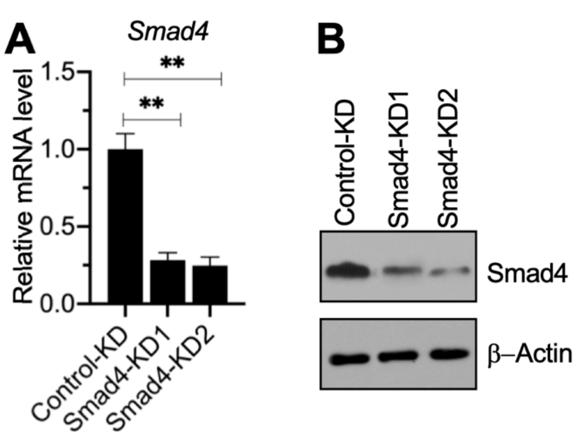

Figure S3. The Smad4 mRNA and protein levels in its knockdown cells

(A) *Smad4* mRNA level. Total RNA samples from Control-KD, Smad4-KD1, and Smad4-KD2 cells were used for RT-qPCR analysis to examine the mRNA level of *Smad4*. $^{**}P < 0.01$. **(B** and **C)** Smad4 protein level. Total cell extracts from cells used in (A) were subjected to immunoblots to examine the protein levels of Smad4 and β-Actin (loading control) **(B)**. The protein signals were quantified using Image J software and then normalized to their corresponding loading controls **(C)**. $^{***}P < 0.001$.

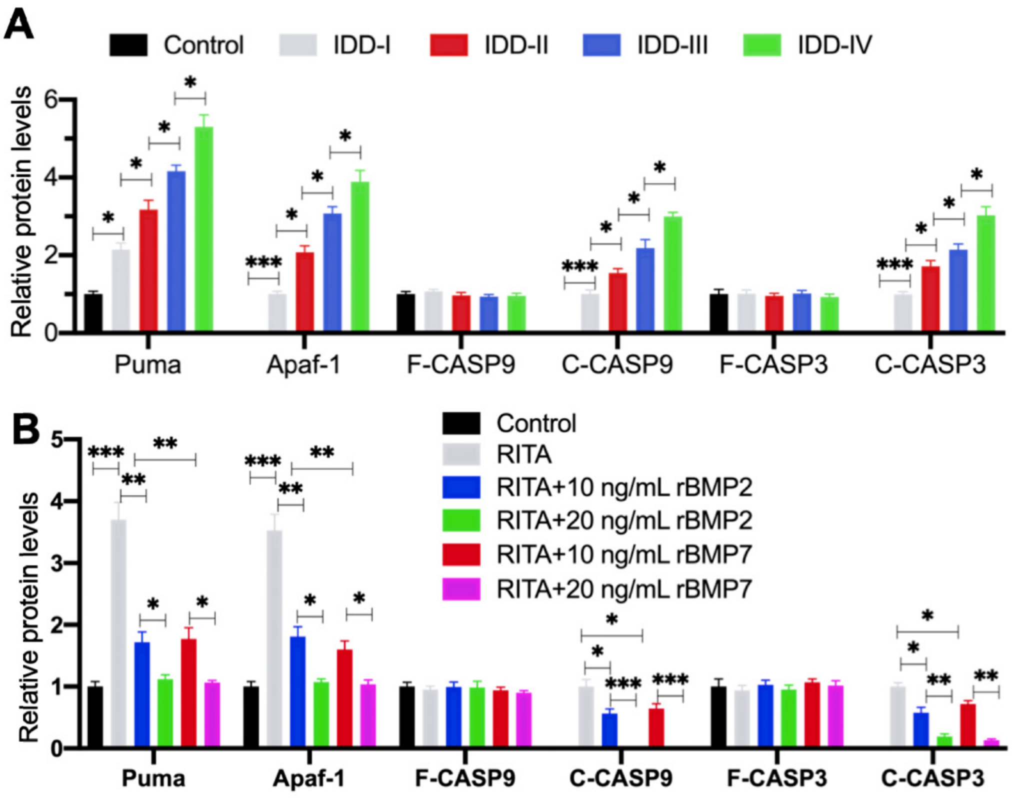

Figure S4. The relative protein levels Puma and its downstream apoptotic molecules

(A) The relative protein levels of Puma and its downstream apoptotic molecules in IDD specimens. The protein bands in Figure 3A were quantified using Image J software and then normalized to their corresponding loading controls. *$P < 0.05$ and ***$P < 0.001$. **(B)** The relative protein levels of Puma and its downstream apoptotic molecules in RITA- and rhBMP-treated cells. The protein bands in Figure 3C were quantified using Image J software and then normalized to their corresponding loading controls. *$P < 0.05$, **$P < 0.01$, and ***$P < 0.001$.

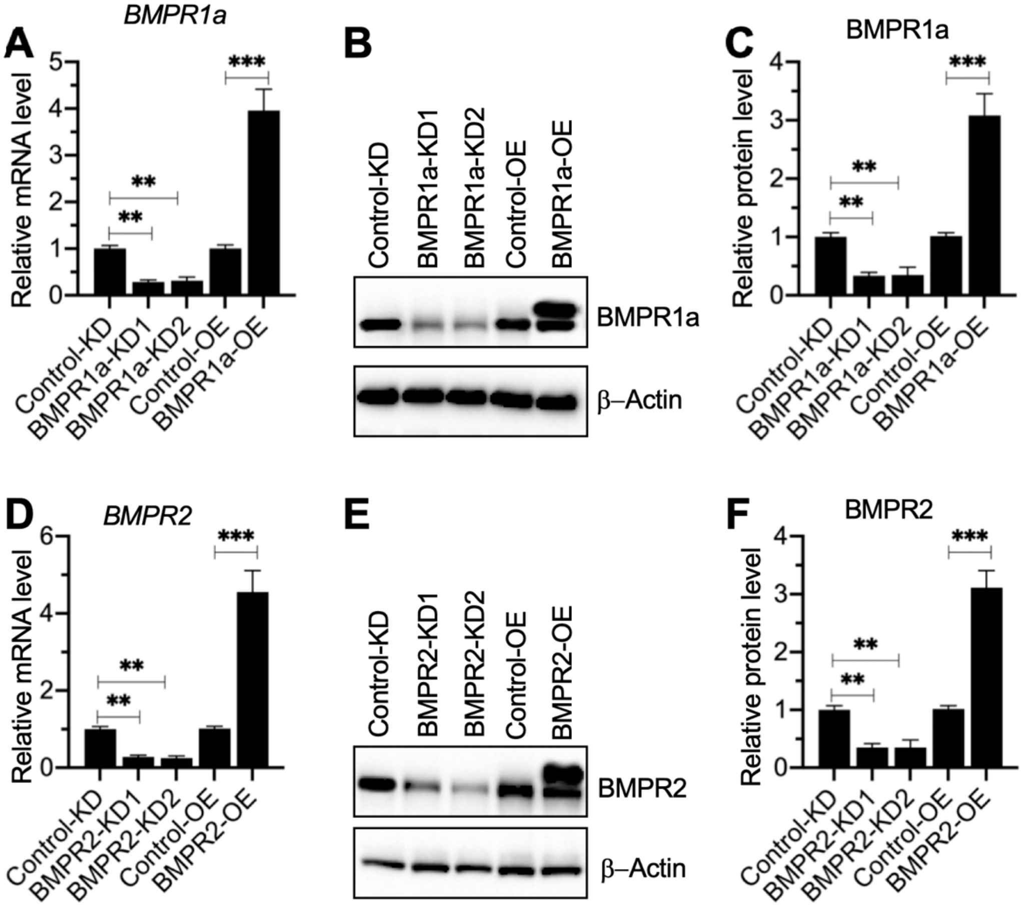

Figure S5. The mRNA and protein levels of BMPR1a and BMPR2 in their corresponding knockdown and overexpression cell lines

(A) *BMPR1a* mRNA level. Total RNA samples from Control-KD, BMPR1a-KD1, BMPR1a-KD2, Control-OE, and BMPR1a-OE cells were used for RT-qPCR analysis to examine the mRNA level of *BMPR1a*. $^{**}P < 0.01$ and $^{***}P < 0.001$. **(B** and **C)** BMPR1a protein level. Total cell extracts from cells used in (A) were subjected to immunoblots to examine the protein levels of BMPR1a and β-Actin (loading control) **(B)**. The protein signals were quantified using Image J software and then normalized to their corresponding loading controls **(C)**. $^{**}P < 0.01$ and $^{***}P < 0.001$. **(D)** *BMPR2* mRNA level. Total RNA samples from Control-KD, BMPR2-KD1, BMPR2-KD2, Control-OE, and BMPR2-OE cells were used for RT-qPCR analysis to examine the mRNA level of *BMPR2*. $^{**}P < 0.01$ and $^{***}P < 0.001$. **(E** and **F)** BMPR2 protein level. Total cell extracts from cells used in (D) were subjected to immunoblots to examine the protein levels of

BMPR1a and β-Actin (loading control) **(E)**. The protein signals were quantified using Image J software and then normalized to their corresponding loading controls **(F)**. ***P* < 0.01 and ****P* < 0.001.

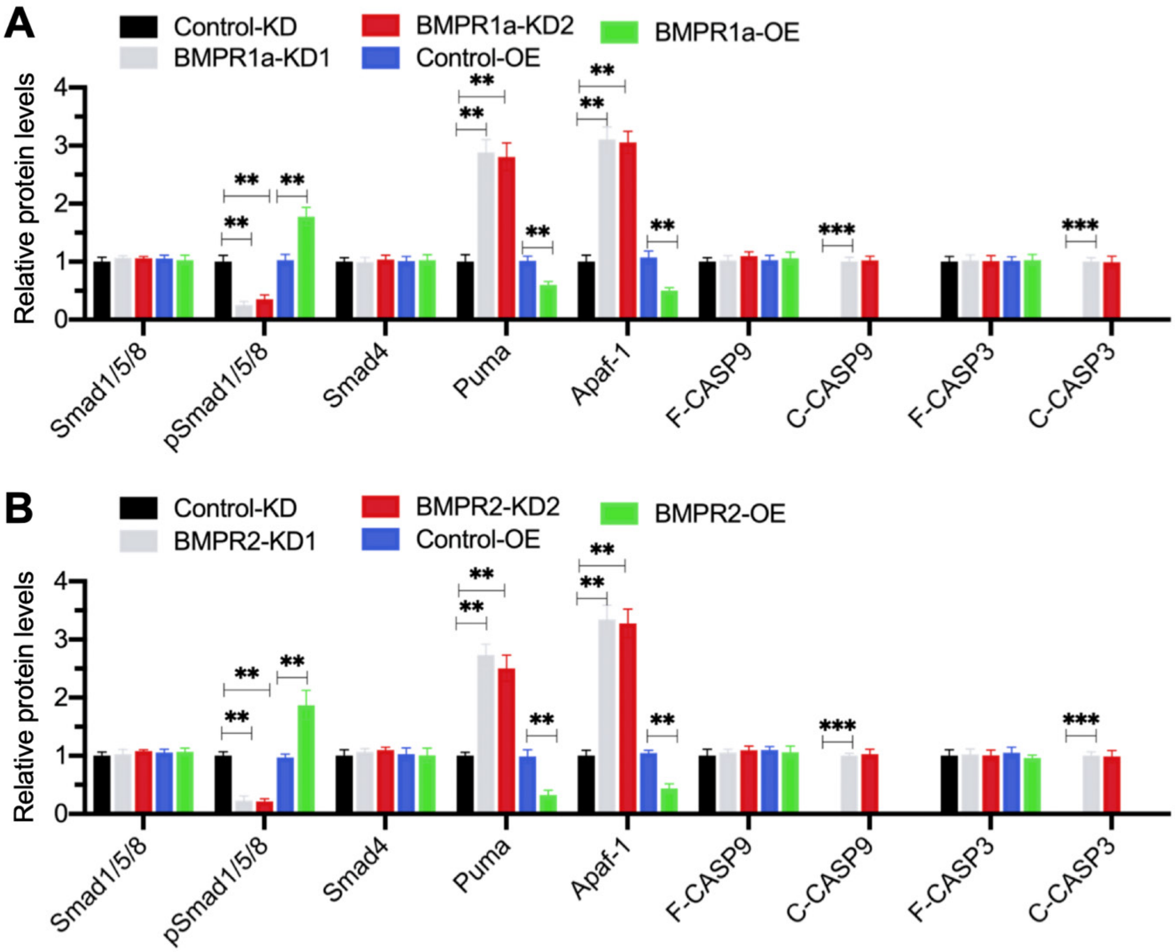

Figure S6. BMP/Smad signaling molecules and Puma-dependent apoptotic molecules in BMPR1a/2-KD and BMPR1a/2-OE cells

(A) The relative protein levels of BMP/Smad signaling molecules and Puma-dependent apoptotic molecules in BMPR1a-KD and BMPR1a-OE cells. The protein bands in Figure 4C were quantified using Image J software and then normalized to their corresponding loading controls. *$P < 0.05$, **$P < 0.01$, and ***$P < 0.001$. **(B)** The relative protein levels of BMP/Smad signaling molecules and Puma-dependent apoptotic molecules in BMPR2-KD and BMPR2-OE cells. The protein bands in Figure 4D were quantified using Image J software and then normalized to their corresponding loading controls. *$P < 0.05$, **$P < 0.01$, and ***$P < 0.001$.

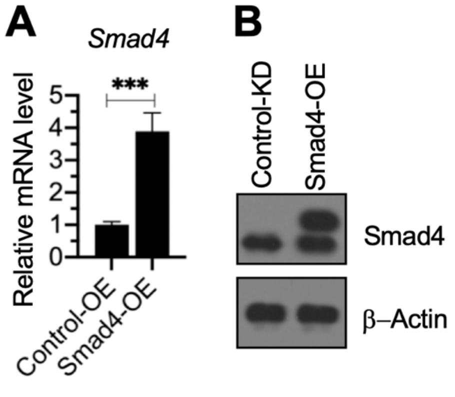

Figure S7. The Smad4 mRNA and protein levels in its overexpression cells

(A) *Smad4* mRNA level. Total RNA samples from Control-OE and Smad4-OE cells were used for RT-qPCR analysis to examine the mRNA level of *Smad4*. ***$P < 0.001$. **(B** and **C)** Smad4 protein level. Total cell extracts from cells used in (A) were subjected to immunoblots to examine the protein levels of Smad4 and β-Actin (loading control) **(B)**. The protein signals were quantified using Image J software and then normalized to their corresponding loading controls **(C)**. ***$P < 0.001$.

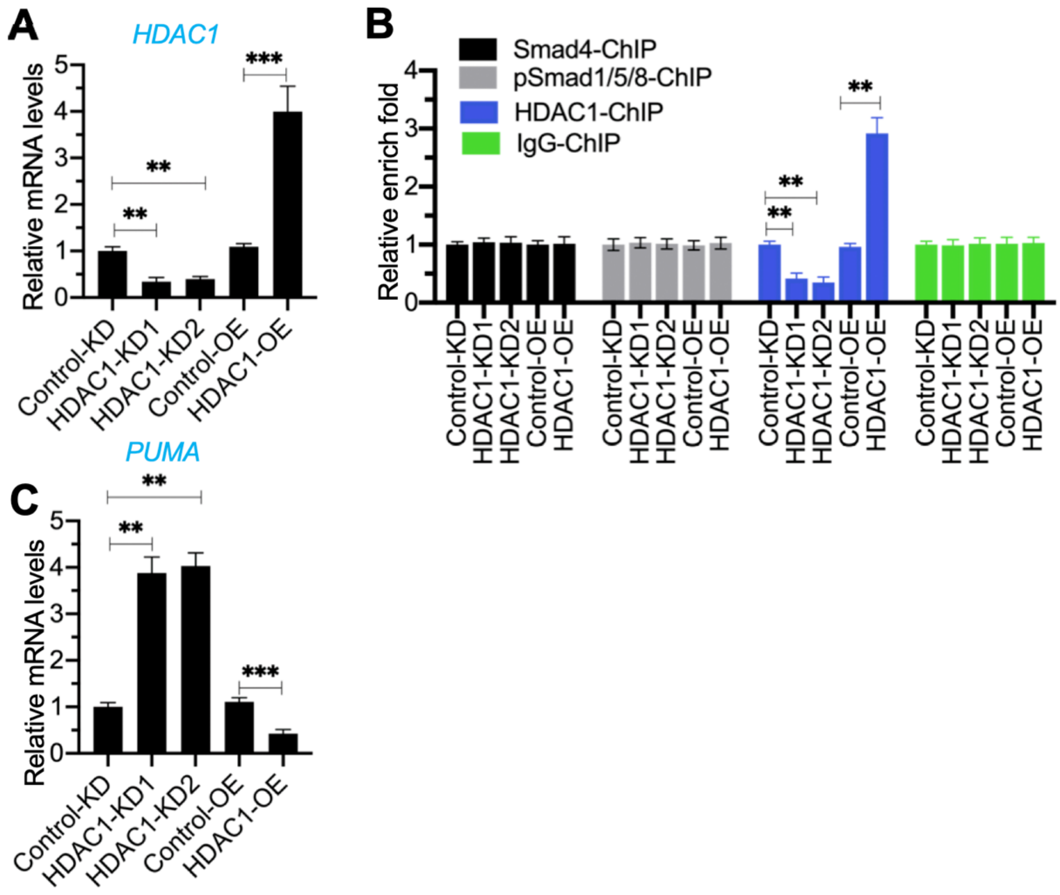

Figure S8. The occupancy of HDAC1-pSmad1/5/8-Smad4 and the expression of *PUMA* in HDAC1-KD and HDAC1-OE cells

(A) *HDAC1* mRNA level. The Control-KD, HDAC1-KD1, HDAC11-KD2, Control-OE, and HDAC1-OE cells were subjected to RT-qPCR assay to detect the expression of *HDAC1*. ** $P < 0.01$ and *** $P < 0.001$. **(B)** ChIP results. Cells used in (A) were subjected to ChIP assays using anti-Smad4, anti-pSmad1/5/8, anti-HDAC1, and IgG, respectively. The input and output DNA samples were subjected to RT-qPCR analysis. ** $P < 0.01$. **(C)** *PUMA* mRNA level. The same RNA samples as in (A) were subjected to RT-qPCR assay to detect the expression of *PUMA*. ** $P < 0.01$ and *** $P < 0.001$.

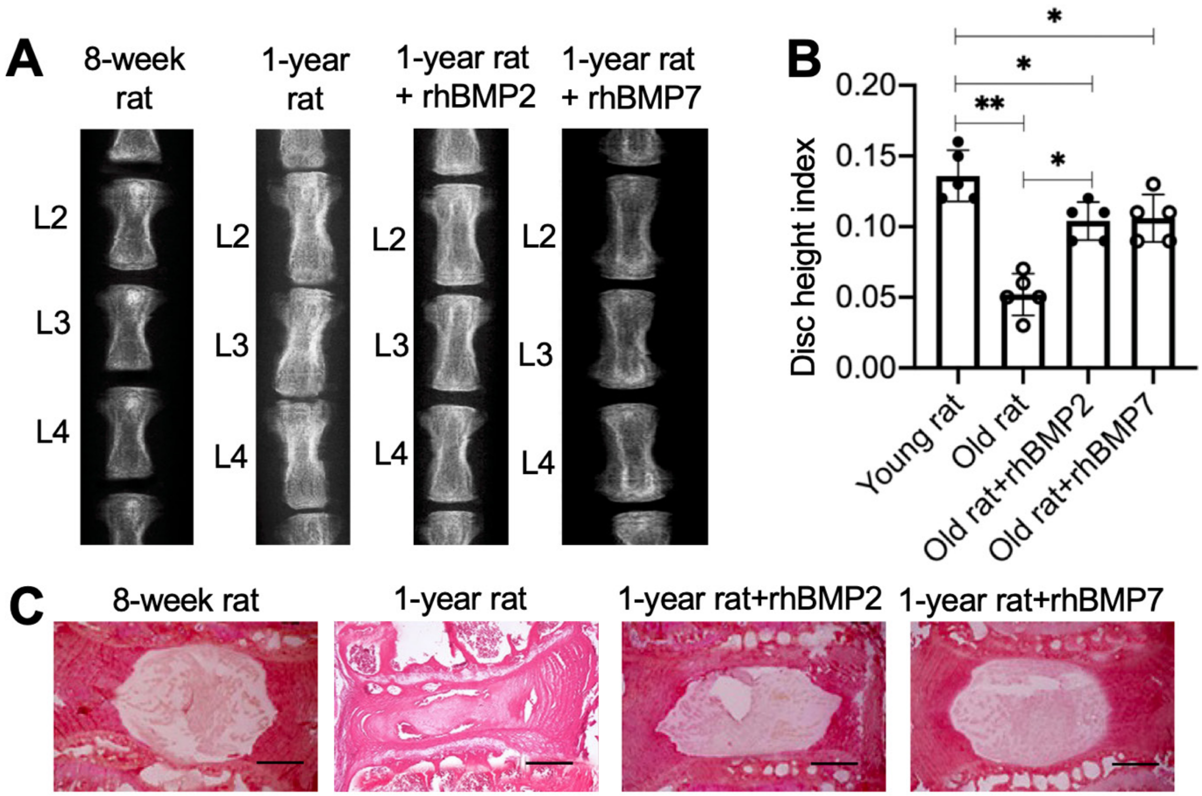

Figure 9. The changes of lumbar IVDs in rats administrated with rhBMPs

(A) X-ray images of lumbar IVDs. Different groups of rats (n=5 for each group) were used for X-ray images, and the representative images of lumbar IVDs (L2-L4) are shown. **(B)** Disc height index (DHI). The DHI was calculated based on lumbar vertebrae. *P < 0.05 and **P < 0.01. **(C)** H&E staining results. The representative H&E staining images of lumbar IVDs from four groups of rats. Bars=200 μm.

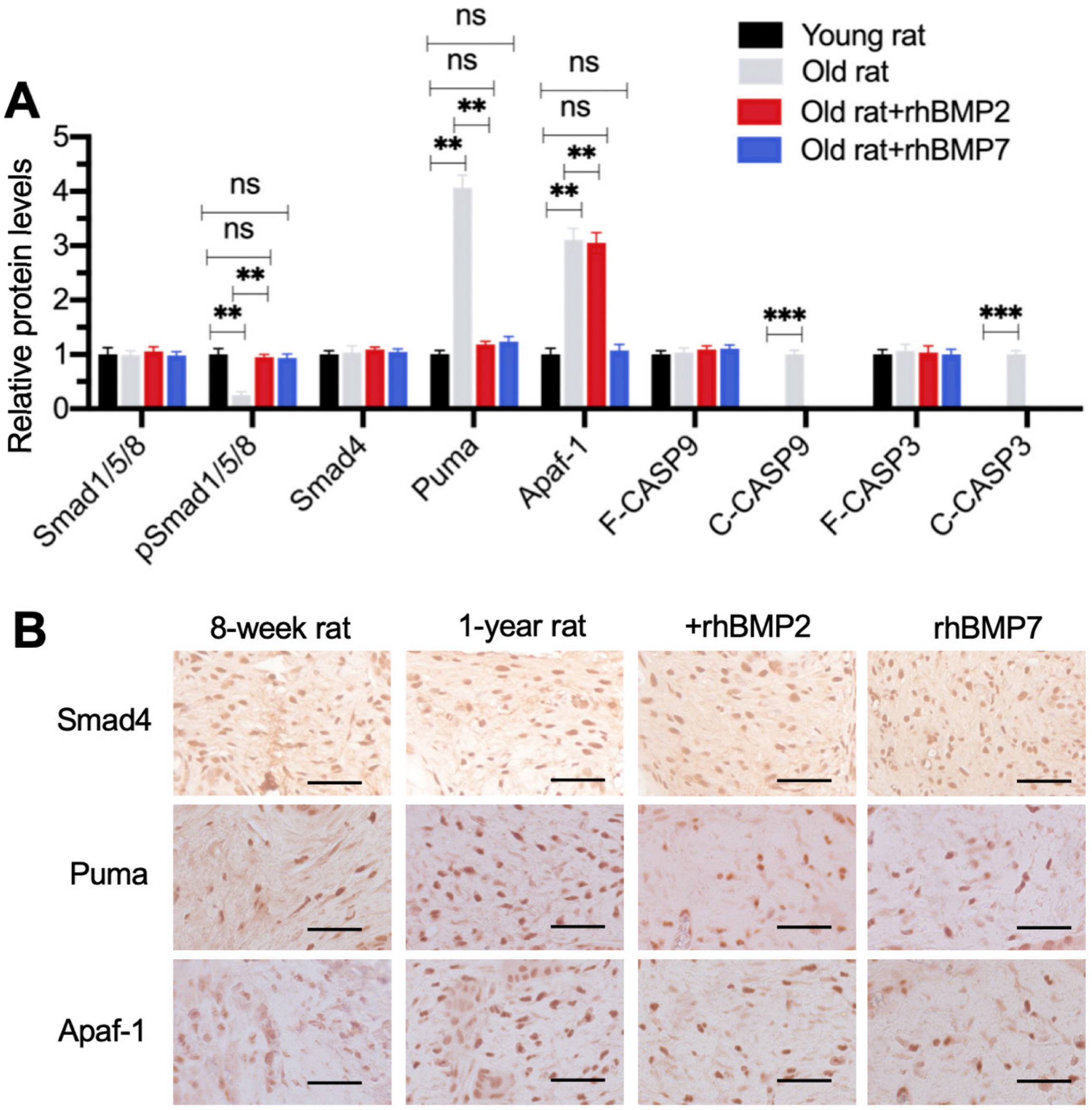

Figure S10. BMP/Smad signaling molecules and Puma-dependent apoptotic molecules in IVDs from rats

(A) The relative protein levels of BMP/Smad signaling molecules and Puma-dependent apoptotic molecules. The protein bands in Figure 7E were quantified using Image J software and then normalized to their corresponding loading controls. ns: no significant difference, $^{**}P < 0.01$, and $^{***}P < 0.001$. **(B)** IHC results. The representative IHC staining images of Smad4, Puma, Apaf-1 in lumbar IVDs from four groups of rats. Bars=20 μm.

Supplementary Table S1. The basic information of participants for blood collection

Participants	Age (years)	Gender (M/F)	Pfirrmann grade
Healthy-1	23	M	N/A
Healthy-2	18	F	N/A
Healthy-3	27	F	N/A
Healthy-4	33	M	N/A
Healthy-5	24	F	N/A
Healthy-6	19	F	N/A
Healthy-7	37	M	N/A
Healthy-8	26	M	N/A
Healthy-9	20	M	N/A
Healthy-10	21	F	N/A
Healthy-11	25	M	N/A
Healthy-12	25	M	N/A
Healthy-13	21	F	N/A
Healthy-14	20	F	N/A
Healthy-15	29	M	N/A
Healthy-16	30	M	N/A
Healthy-17	28	F	N/A
Healthy-18	26	F	N/A
Healthy-19	21	F	N/A
Healthy-20	25	M	N/A
IDD-1	57	F	IV
IDD-2	68	F	IV
IDD-3	72	F	IV
IDD-3	66	M	IV
IDD-4	68	M	IV
IDD-5	63	F	IV
IDD-6	78	M	IV

IDD-7	58	M	IV
IDD-8	71	F	IV
IDD-9	69	M	IV
IDD-10	71	M	IV
IDD-11	74	F	IV
IDD-12	66	M	IV
IDD-13	73	F	IV
IDD-14	75	M	IV
IDD-15	69	F	IV
IDD-16	71	F	IV
IDD-17	65	M	IV
IDD-18	69	F	IV
IDD-19	72	M	IV
IDD-20	70	F	IV

Supplementary Table S2. The basic information of participants for IVD collection

Participants	Age (years)	Gender (M/F)	Pfirrmann grade
Control	20	M	N/A
IDD-1	66	M	I
IDD-2	68	M	II
IDD-3	70	M	III
IDD-4	73	M	IV

Supplementary Table-S3. Primers for vector construction

Vectors	Forward primers	Reverse primers
pCDNA3-2×Flag-Smad4	CGGGATCCATGGACAATATGTCTATTACG	CCGCTCGAGTCAGTCTAAAGGTTGTGGGT
pCDNA3-2×Flag-BMPR1a	CGGGATCCATGCCTCAGCTATACATT	CCGCTCGAGTCAGATTTTTACATCTTGG
pCDNA3-2×Flag-BMPR2	CGGGATCCATGACTTCCTCGCTGCAGC	CCGCTCGAGTCACAGACAGTTCATTCCTAT
pCDNA3-2×Flag-Puma	CGGGATCCATGGCCCGCGCACGCCAGGA	CCGCTCGAGCTAATTGGGCTCCATCTC
pCDNA3-Myc-HDAC1	CGGGATCCATGGCGCAGACGCAGGGCA	CCGCTCGAGTCAGGCCAACTTGACCT
pGL4.26-PUMA^{WT}	CGAGCTCGCTGAGGCAGAAGACTTG	CCGCTCGAGGTCTCAGGCCGCCCGGC
pGL4.26-PUMA^{Mut1}	CTGTGGCCTTGTAGACGTGAGT	ACTCACGTCTACAAGGCCACAG
pGL4.26-PUMA^{Mut2}	GCCCGTCGGTCGAGACGTGTACGC	GCGTACACGTCTCGACCGACGGGC

Supplementary Table-S4. Primers used for RT-qPCR analysis

Gene	Forward (5'-3')	Reverse (5'-3')
Smad4	TCAGTGTCATCGACAGATG	GACTGATAGCTGGAGCTA
PUMA	GGATGGCGGACGACCTCAACG	GAGTCCCATGATGAGATTG
KLF17	GATCTCAGGACTCTCTTGTCA	CTACCAGTCCCTTCCTGAG
TGM2	CAGCTACCTGCTGGCTGAG	AGGGAGCGGGTTCTGCAG
COL1A1	TACATGGACCAGCAGACTG	GTGCAGCCATCGACAGT
AXIN1	GACATGGAGCTCTCCGAGA	CTCACCAGGGTGCGGTAG
XPO1	ATGGTACAGAGTGGTCATGG	TGCCTCTTTTCTGTTCAC
BMPR1a	TGCTCATCGAGACCTAAAG	GGTATTCAAGGGCACATCA
BMPR2	TATCAGCAAGACCTTGGGA	ACAAGATTTATGTCCCCTT
HDAC1	CTGCCTATGCTGATGCTG	AGTAGTCATTGTATGGAAG
β-Actin	TACGAGCTGCCTGACGGCCA	AGACAGCACTGTGTTGGCG

Supplementary Table-S5. Differentially expressed genes dependent on Smad4

Genes	Control	Smad4-KD1	Smad4-KD2
PUMA	1.7	9.4	10.5
KLF2	1.4	3.1	2.5
KLF4	2.1	4.5	5.6
PAX1	1.6	4.3	2.6
MEN1	2.1	4.3	3.5
PMS2	1.5	2.1	2.5
CXCL8	1.8	4	3.4
SKIL	2.1	4.5	3.2
EFNA1	1.5	3.2	3.7
KLF17	1.7	2.1	4.2
CXXC5	1.1	3.2	2.6
EDN1	2.1	4.3	4.6
ETS2	1.5	2.6	3.2
TGIF	1.1	2.3	3.2
TGM2	1.3	3.4	2.5
LTBP2	1.3	2.3	3.1
ITGA2	2.2	4.3	3.5
LAMC2	1.6	2.3	3.5
CCND1	1.1	3.2	2.4
MSH6	1.6	3.4	4.2
CDKN1A	1.5	3.2	3.5
COL1A1	-1.5	-4.5	-4.3
CSSX2	-2.2	-3.2	-5.5
EID2	-2.1	-7.5	-8.2
CCN2	-3.2	-5.1	-6.9
KRT19	-2.5	-4.1	-5.4
AXIN1	-5.4	-7.2	-9.4
ITGB1	-1.4	-4.3	-3.2
TIMP3	-2.1	-5.4	-4.1
SNW1	-4.2	-7.2	-8.3
CTNNA1	-2.6	-4	-4.7
XPO1	-1.18	-3.3	-2.4

Supplementary Table-S6. The Smad4-associated proteins by MS analysis

Protein	Protein description	Molecular weight (Da)	MASCOT scores
Smad4	Mothers Against Decapentaplegic Homolog 4	60439	2102
Smad5	Mothers Against Decapentaplegic Homolog 5	52258	2056
Smad1	Mothers Against Decapentaplegic Homolog 1	52260	2022
Smad8	Mothers Against Decapentaplegic Homolog 8	52493	1958
HDAC1	Histone Deacetylase 1	55103	1951
TRIM33	Tripartite Motif Containing 33	122533	1921
AKT1	AKT Serine/Threonine Kinase 1	55686	1887
FOXH1	Forkhead Box H1	39257	1843
CDKN1A	Cyclin Dependent Kinase Inhibitor 1A	18119	1778
MSH6	MutS Homolog 6	152786	1725
RNF111	Ring Finger Protein 111	108862	1709
RBPMS	RNA-Binding Protein With Multiple Splicing	21802	1693
BUB1	BUB1 Mitotic Checkpoint Serine/Threonine Kinase	122375	1655
ZMIZ1	Zinc Finger MIZ-Type Containing 1	115483	1612
SNAI1	Snail Family Transcriptional Repressor 1	29083	1588
CAV1	Caveolin 1	20472	1523
RPS6KB1	Ribosomal Protein S6 Kinase B1	59140	1427
HIPK2	Homeodomain Interacting Protein Kinase 2	130966	1155
RANBP3L	RAN Binding Protein 3 Like	52211	1076
CTDSP2	CTD Small Phosphatase 2	30664	995
DVL1	Dishevelled Segment Polarity Protein 1	75187	901
UBE2D1	Ubiquitin Conjugating Enzyme E2 D1	16602	832
SOX9	SRY-Box Transcription Factor 9	56137	767
GARS1	Glycyl-TRNA Synthetase 1	83166	743
IKZF1	IKAROS Family Zinc Finger 1	57528	701

BRD4	Bromodomain Containing 4	152219	678
MBD2	Methyl-CpG Binding Domain Protein 2	43255	634
IFFO1	ntermediate Filament Family Orphan 1	61979	602
CHD4	Chromodomain Helicase DNA Binding Protein 4	218005	554
TAPBPL	TAP Binding Protein Like	50183	524
SRRM2	Serine/Arginine Repetitive Matrix 2	299615	503
DDX23	DEAD-Box Helicase 23	95583	501
